# Computational construction and design optimization of a novel tri-tube heart valve

**DOI:** 10.1007/s10237-025-01956-5

**Published:** 2025-05-26

**Authors:** Jirong Li, Yijiang Yu, Robert T. Tranquillo

**Affiliations:** 1 Department of Biomedical Engineering, University of Minnesota, 7-114 NHH, 312 Church St SE, Minneapolis, MN 55455 USA; 2 Department of Mechanical Engineering, University of Minnesota, Minneapolis, MN USA; 3 Department of Chemical Engineering and Materials Science, University of Minnesota, Minneapolis, MN USA

**Keywords:** Heart valve, Coaptation, Washout, Optimization, Finite element analysis, Fluid–structure interaction

## Abstract

A finite-element-based algorithm for the in silico construction of a novel tri-tube heart valve was developed to facilitate optimization of the leaflet geometry. An anisotropic hyperelastic model fitted to high-strain rate planar equibiaxial tension and compression data was used to approximate the nonlinear and anisotropic material behavior of biologically-engineered tubes and simulate valve closure under steady back pressure and steady forward flow. Four metrics were considered to evaluate valve performance in simulated closure: coaptation area, regurgitation area, pinwheel index, and prolapse area. Response surfaces revealed competing objectives between metrics for a valve of target 24 mm diameter in terms of two design parameters, tube diameter and leaflet height. A multi-objective genetic algorithm determined an intermediate tube diameter and leaflet height (16 mm and 11 mm, respectively) of the design space as optimal. Additionally, steady flow simulations were performed using two-way fluid–structure interaction with selected designs to examine washout behind leaflets with particle tracking. One design close to the optimal point for valve closure indicated washout for particles initially distributed behind leaflets. Though comprehensive valve design optimization requires flow analysis over multiple valve cycles to capture all effects associated with flow, this methodology based on diastolic state geometry optimization followed by steady washout analysis reduces the space of design variables for further optimization.

**Supplementary Information:**

The online version contains supplementary material available at 10.1007/s10237-025-01956-5.

## Introduction

A goal of heart valve tissue engineering is to create a valve that provides mechanical function while being biocompatible and hemocompatible (Fioretta et al. 2018). The geometrical design of a normal native valve is inherently optimized with respect to the unique features of normal tissue. The resulting solid stresses are connected to the homeostatic *versus* pathological (e.g. pro-calcifying) phenotype of the resident valvular interstitial cells (Sakamoto et al. 2017; Zhong et al. 2018). Likewise, the dynamic shape of the valve during cardiac cycling determines shear stresses on the leaflet surfaces that determine whether the blood-contacting endothelial cells are in a pro- *versus* anti-inflammatory/thrombogenic phenotype (Baeyens et al. 2016; Davies et al. 2013; Roux et al. 2020; Zhou et al. 2014).

Using specific constitutive equations for the valve material (e.g. tissue, polymer) and fluid (e.g. blood, water) along with the known geometry of either a normal native valve or a prosthetic valve, the fluid–structure interaction (FSI) hydrodynamics problem can be solved computationally to predict valve function under a specified inlet pressure or velocity boundary condition. Previous studies used isotropic hyperelastic material models to study prosthetic heart valves with polymer leaflets (Oks et al. 2022; Sigüenza et al. 2018; Spühler et al. 2018) or bioprosthetic heart valves (BHV) (Hsu et al. 2014, 2015; Kamensky et al. 2017). For a more precise description of the anisotropic tissue behavior in tissue leaflets associated with commissure-to-commissure collagen fiber alignment, Wu et al. (2018) used the anisotropic Lee-Sacks model to reproduce the mechanical behavior of a BHV made of cross-linked bovine pericardial tissues with high fidelity. Jafar et al. (2020) used Guccione’s anisotropic material model to assess the effect of asymmetric valve annulus configurations on the coaptation of a BHV under applied back pressure. Lee et al. (2020) applied a Holzapfel-type anisotropic hyperelastic model of leaflets to simulate a BHV during cardiac cycling using FSI, where hydrodynamics and valve shape changes showed good agreement with data obtained from a pulse duplicator. They further extended the material model to investigate leaflet fluttering during systole with different valve designs by tuning leaflet thickness and valve geometry, where a smaller diameter valve and larger leaflet thickness yield higher fluttering frequencies, independent of flow conditions (Lee et al. 2021). Kaiser and coworkers simulated the native leaflet anisotropy using a fiber-based model, in which 1D fibers cover the leaflet surface from commissure to commissure as a collection of nonlinear springs (Kaiser et al. 2021, 2023). Though they did not directly model the material properties of the leaflet, the FSI results are comparable to experimental data. Other relevant studies include (De Hart et al. 2003; Ge and Sotiropoulos 2010) and are reviewed in (Y. Wu et al. 2024; Zakerzadeh et al. 2017).

A rational approach to identifying valve designs that should meet performance metrics is computer-aided design (CAD). With CAD, the valve geometry can be tuned, followed by subsequent FSI analysis. Optimization of BHV geometry has been investigated. Hsu and coworkers (2014, 2015) developed a framework to design a stented valve geometry on an interactive CAD platform, allowing rapid leaflet generation of a spline leaflet surface based on input design parameters. In (Hsu et al. 2015), designs were screened with multiple material models for simulations using physiological pressure boundary conditions, where the leaflet shapes from structural-mechanical and FSI simulations were compared and discussed. This design framework was adopted and further refined (Kamensky et al. 2017; Xu et al. 2018). Specifically, Xu et al. (2018) formulated a framework for patient-specific BHV design by extracting aortic root geometry from medical images with splines defining the free edges and radial “belly curves” of the leaflets for parameterization. The coaptation area of each design during FSI was evaluated and screened as an optimization strategy.

In the case of heart valve tissue engineering, CAD-based optimization creates both an opportunity and challenge since the geometrical design is not specified and thus is inherently an optimization problem. It is a complex optimization problem due to multiple competing objectives, such as maximized coaptation and minimized pinwheeling, associated with larger and smaller leaflet area, respectively. It is also subject to constraints, such as the target valve diameter. It is further complicated by the fact such optimization requires solving the computationally demanding FSI problem. However, before the assessment of designs based on hydro/hemodynamic performance that entails FSI, an assessment of designs that consider critical metrics in the absence of flow could be conducted to identify a subset of designs (or design variable space) that merits the computational cost of FSI solutions, as a two-step optimization framework. During diastole, the principal forces acting on the leaflets from surrounding fluid can be approximated as a uniformly distributed pressure load (Zakerzadeh et al. 2017). Therefore, only designs that yield adequate coaptation and minimal pinwheeling, for example, with a steady back-pressure (diastolic gradient) should be considered further with FSI analysis. It is this two-stage optimization procedure that we have adopted in this study as a combination of structural-mechanical and FSI simulations. Accordingly, we consider the first stage of valve geometry optimization under a diastolic pressure gradient, and then we illustrate how different design points with the same leaflet area exhibit different steady flow behavior with a focus on washout behind the leaflets.

**Fig. 1:**
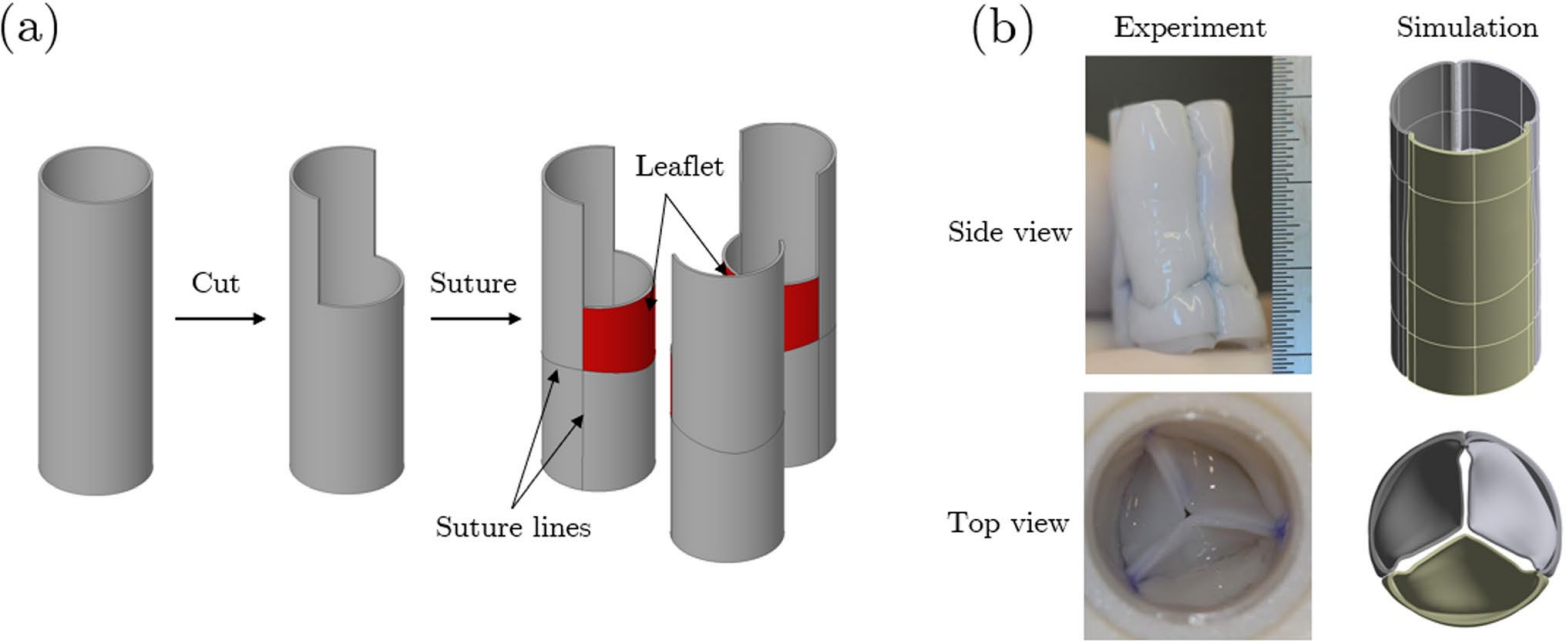
**a** Experimental construction of the tri-tube tissue-engineered heart valve (TEHV) by suturing three biologically-engineered tubes together. **b** Comparison between an experimental TEHV and a TEHV constructed and simulated in silico. Figure from (Syedain et al. 2021) reproduced with permission

The specific valve design to be optimized here is a novel tri-tube valve where three tubes are sutured together in a closed ring after cutting out a section from the end of each tube, and then the bottom of each tube is sutured closed to form a leaflet, as shown by the hatched region in Fig. 1 a (Syedain et al. 2021). This tissue-engineered heart valve (TEHV) yields a wide range of possible leaflet sizes and shapes for a target valve diameter because three tubes of any diameter beyond some minimum, for a chosen leaflet height, can be sewn together into a coapting valve of target diameter (noting tubes of smaller diameter yield less leaflet area, and vice versa). This leads to an interesting design optimization problem that dictates valve performance, at least at implantation, in a reasonably predictive manner given the measurable constitutive mechanical properties of the tubes of circumferentially-aligned collagenous matrix grown in vitro from donor fibroblasts (Syedain et al. 2016, 2017). Such engineered biomaterials do not recapitulate the constitutive mechanical behavior of valve tissue because they do not possess the unique combination of architecture, microstructure, and composition of natural tissue. Thus, to achieve a similar mechanical function of a native valve based on metrics such as peak pressure drop or closing volume, a valve made from an engineered material likely entails different values of geometrical parameters, such as the leaflet height, shape, and area. Superimposed on mechanical function metrics are considerations related to biocompatibility and hemocompatibility, such as washout time of the sinus that can correlate to clotting propensity or adverse shear stress waveforms on the outflow surface of the leaflet and adjacent valve root that correlate to endothelial activation.

The proposed two-stage optimization first requires a finite-element-based simulation to reproduce the valve construction process that sutures three tubes together, as described above (Fig. 1), to rigorously define the leaflet geometry. A simpler application of such valve fabrication in silico was previously described for a tubular valve comprised of a single synthetic tube in order to computationally define the three commissures (Sodhani et al. 2018); the deformed configuration was then imported into an FSI code as the stress-free state of the “stitched valve”. In contrast, the tri-tube valve construction is substantially more complex. Before performing FSI, we will optimize the leaflet geometry based on the aforementioned multiple objectives resulting from a constant diastolic pressure gradient.

The tri-tube valve was conceived to provide stable commissures during valve growth for pediatric pulmonary applications, notably right ventricular outflow tract reconstruction for the repair of tetralogy of Fallot (Syedain et al. 2021). However, an adult-size pulmonary valved conduit made from this tri-tube valve by suturing it within a longer tube of the same material (Syedain et al. 2025) is a potential source for a pulmonary valved conduit that is needed during a Ross procedure for a young adult, where the patient’s pulmonary valve is transplanted to the aortic position as a replacement valve and provides superior outcomes (Yokoyama et al. 2023). Thus, we have set the target valve diameter in this study to be 24 mm, and somatic valve growth post-implantation is irrelevant for its optimization.

The rest of this presentation is organized as follows: In Sect. 2, we outline the material characterization of engineered tissue and a detailed two-stage optimization framework for the TEHV, including the setup for valve construction, closure, and FSI simulation. In Sect. 3, we present the mechanical testing and the valve closure simulation results, along with validation cases for the model. The measured metrics between different designs are fed to optimization tools to deliver an optimal design for TEHV, followed by FSI simulations for washout examination. Those results are discussed in Sect. 4 and concluding remarks are made in Sect. 5.

## Methods

### Tube mechanical characterization

The biologically-engineered tubes used to construct the valve are comprised of an acellular hydrated collagenous matrix that is aligned in the circumferential direction, exhibiting both anisotropic and nonlinear tensile mechanical properties (Syedain et al. 2021). Because the in silico valve construction and function simulations entail tube bending, both unconfined compression and planar equibiaxial tension testing were conducted to fit relevant strain energy laws for these simulations.

#### Unconfined compression testing

The bulk compressive properties of tubes were measured in an unconfined compression test using a rheometer (RSA-G2 Solids Analyzer, TA Instruments) at room temperature. A parallel plate fixture of 8-mm diameter was employed. A 4 × 4 mm square flat piece of the biologically-engineered tube grown from ovine dermal fibroblasts as previously reported (Syedain et al. 2016, 2017) was placed in between the upper and lower plates, almost covering the plates. The upper plate was moved vertically at a constant displacement rate of 3%/s to generate uniaxial compression, and the test was run until the strain reached ~ 30%. The average of 5 compression tests on a representative sample was determined. These data were used along with equibiaxial tension data in fitting the strain energy laws as discussed next.

#### Planar biaxial tension testing

The planar biaxial tensile properties of tubes were measured on an Instron-Sacks testing system. Five cruciform-shaped samples cut from biologically-engineered tubes spanning 3 separate production batches were tested. Samples were clamped with grips (width 10 mm), preloaded to 0.005N tension, and stretched equibiaxially to ~ 30% grip strain for 30 cycles at the highest achievable displacement rate of 4.75 mm/s (≈75%/s) preconditioning. 30 cycles sufficed to achieve a stable peak force. Samples were then stretched under actuator displacement control at the same rate to a grip strain of ≈25% was reached. The initial width/thickness and length/thickness of cruciform arms from preload images were used to calculate the cross-sectional area for engineering stress in circumferential and radial directions. The unloaded thickness of each specimen was measured using digital calipers. A speckle tracking method previously detailed (Raghupathy 2011) was used to measure true strain in the central 5 × 5 mm region of the cruciform: Speckles were placed on the specimen using dried Verhoeff’s stain. High-resolution video during the testing was recorded using a Canon Eos Rebel T3i camera with 100 mm f2.8 macro lens. Digital image correlation using the iterative least-squares method with the zero-normalized cross correlation function was used to track speckles. Strain was computed from nodal displacements of the associated mesh using the finite-element theory of bilinear quadrilateral mesh elements.

#### Material models

An anisotropic hyperelastic (AHYPER) material model defined through strain energy density function was used to capture the material’s nonlinearity and anisotropy in tension (ANSYS Inc 2022b). The isotropic part of the AHYPER strain-energy function has a third-order reduced polynomial to describe the isotropic contribution of the non-fiber extracellular matrix. The anisotropy due to fibers oriented along two principal directions, in general, is accounted for in AHPER by this exponential-function-based energy function:

1 $${\text{W}} = \mathop \sum \limits_{i = 1}^{3} a_{i} \left( {I_{1} - 3} \right)^{i} + \frac{{c_{1} }}{{2c_{2} }}\left[ {\exp \left( {c_{2} \left( {I_{4} - 1} \right)^{2} - 1} \right)} \right] + \frac{{e_{1} }}{{2e_{2} }}\left[ {\exp \left( {e_{2} \left( {I_{6} - 1} \right)^{2} - 1} \right)} \right] + \frac{1}{d}\left( {J - 1} \right)^{2}$$

where $I_{1} = \lambda_{1}^{2} + \lambda_{2}^{2} + \lambda_{3}^{2}$ is the first principal strain invariant of the right Cauchy-Green deformation tensor as a function of principal stretch ratios $\lambda_{i}$, $a_{i}$ are the coefficients of the isotropic third-order polynomial function, $I_{4} = \lambda_{1}^{2}$ and $I_{6} = \lambda_{2}^{2}$ are the isochoric stretch ratios for two fiber families, $c_{i} \text{ and } e_{i}$ are material parameters specifying the fiber stiffness in each principal direction. The fiber directions in each element are specified initially (circumferential and axial in the cylindrical coordinate system of the tube used to construct the valve) and change based on the local deformation throughout the simulation. Previous studies have shown that the engineered tubes possess circumferentially aligned collagen fibrils (Syedain et al. 2021). As described in the following section, the flattened tubes were sutured parallel to each other to form a ring. Consequently, we assumed the constructed valve retained its circumferential alignment, which was later verified in the results. $d$ is the material incompressibility parameter inversely proportional to the bulk modulus, and $J = \lambda_{1} \lambda_{2} \lambda_{3}$ is the determinant of the deformation gradient, equal to the volume ratio. The material constants in the AHYPER model were estimated via least-squares regression of experimental stress–strain data. Given the nature of the valve construction from the tubes (Fig. 1a), the same model fit obtained using MCalibration (Ansys Inc., Canonsburg, PA, USA) applies to both the valve root and leaflets.

In addition, we applied an isotropic HYPERFOAM model (Ogden 1972, 1976) for the in silico valve construction procedure where compressive strains of greater magnitude occur as discussed subsequently and a more accurate fit than AHYPER was desired. This strain energy density function equation is given by:

2 $$W = \frac{\mu }{\alpha }\left( {J^{{\frac{\alpha }{3}}} \left( {\overline{{\lambda_{1} }}^{\alpha } + \overline{{\lambda_{2} }}^{\alpha } + \overline{{\lambda_{3} }}^{\alpha } } \right) - 3} \right) + \frac{\mu }{\alpha \beta }\left( {J^{ - \alpha \beta } - 1} \right),$$

where $\overline{{\lambda_{1} }}^{\alpha } , \overline{{\lambda_{2} }}^{\alpha } and \overline{{\lambda_{3} }}^{\alpha }$ are the principal stretch ratios, $J$ is the deformation gradient, $\mu$ denotes shear modulus, $\alpha \text{ and } \beta$ are the material constants associated with bulk modulus, which can greatly differ in compression versus tension in this model.

### Valve construction simulation

**Fig. 2:**
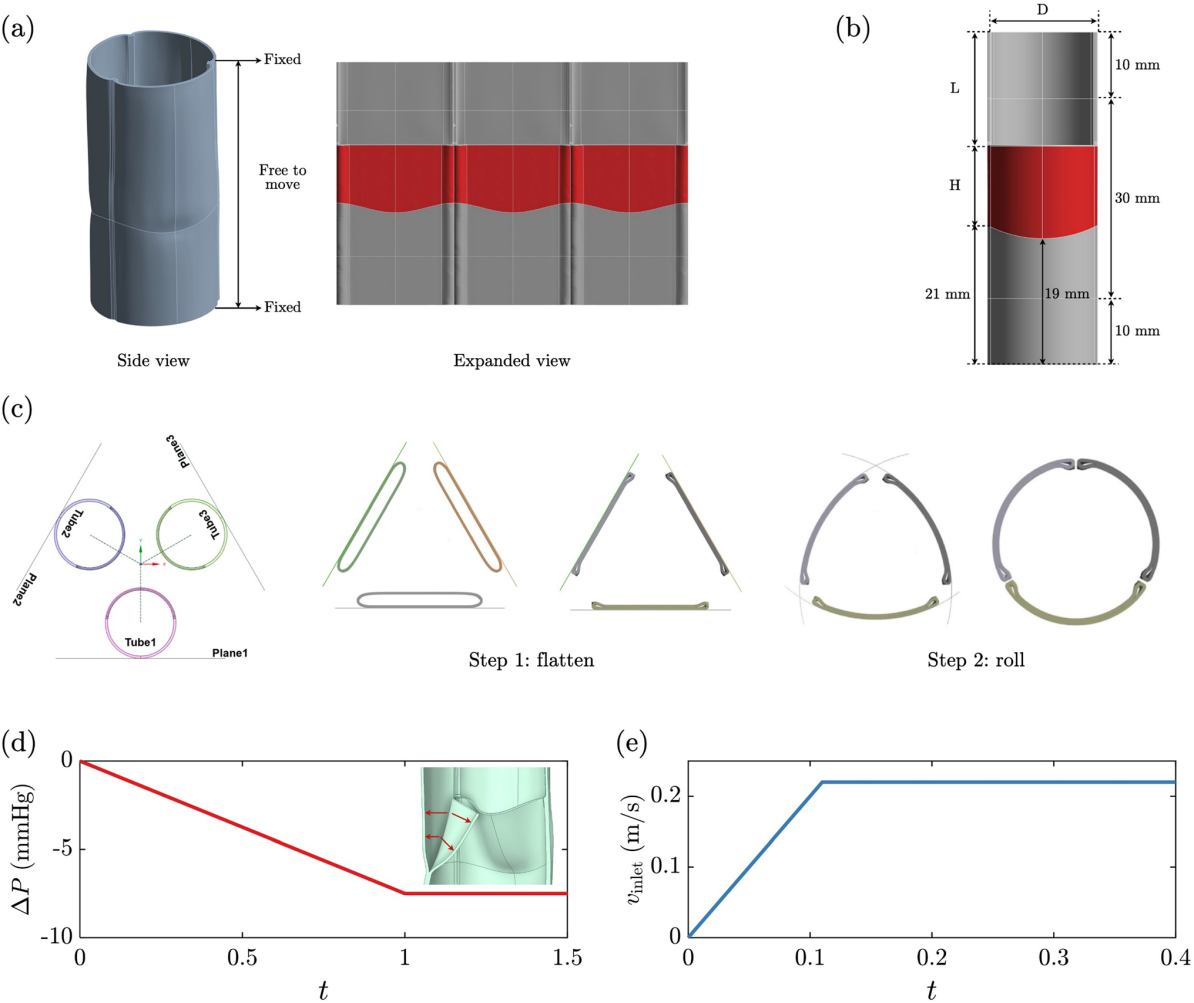
Tri-tube valve definition, in silico construction, and simulation conditions. **a** The top and bottom edges of the conduit are fixed while the material in between is free to move from applied pressure. Portions of the tubes colored red that become the three leaflets in the valve are seen in the expanded view. **b** Dimension of a single tube with diameter D and height of 50 mm. L is the amount of material needed to be removed to define the leaflet height H given the fixed position of the leaflet base (19 mm above the bottom edge). The red shaded area is the resulting leaflet. The valve geometry resulting from construction is determined entirely by H and D and the constitutive model. **c** Top view of two-step valve construction. Step 1: each tube is pushed toward a construction plane oriented as shown, then the tubes are flattened by a prescribed pressure simulating the effect of gravity observed during suturing on the lab bench. Step 2: the three planes are progressively bent to form a closed ring of the tubes with the target valve diameter (see Supplementary material 1 for animation). **d** Pressure profile applied uniformly to the outflow surfaces of the leaflets and outflow segment of the root during the valve closure simulation under sustained back pressure. The inset figure shows pressure acts normal on the valve surfaces. **e** Velocity inlet waveform used in the FSI steady forward flow simulation

A tri-leaflet valve with 24 mm diameter, relevant for adult aortic or pulmonary valve replacement, and 30 mm height was constructed with three identical biologically engineered tubes by connecting the tubes to form a closed ring, simulating the suturing process performed in the lab using finite element method with the HYPERFOAM model. The expanded view of the resulting closed-ring configuration in Fig. 2a shows the three connected tubes and associated leaflets (red-shaded regions). The details of the fabrication process are described subsequently.

The geometry of the resulting tri-leaflet valve in Fig. 2a is determined by the tube dimensions illustrated in Fig. 2b. The tube has a height of 50 mm and a diameter $D$ that can be varied for optimization, discussed subsequently. Two 10 mm sections at the top and bottom mimic the conduit connecting distal and proximal to the native artery. To create a leaflet with a height $H$, a section of tube material is cut and removed. The amount is determined by the arc length $c\left( {c = \pi D - 24\pi /3} \right)$ that achieves a valve diameter of 24 mm, and a vertical length $L$. Since the distance between the suture line closing the tube and the bottom of the tube is fixed (21 mm from the two ends of the suture line to the tube end, and 19 mm from the center of the suture line to the tube end), $H$ is tuned by varying $L\left( {H = 50 - 21 - L} \right)$. Therefore, a leaflet area (LA) can be calculated based on D and H, shown as the shaded area in Fig. 2a. The leaflet height $\left( {7 mm \le H \le 13.25 mm} \right)$ and the tube diameter $\left( {15 mm \le D \le 16.875 mm} \right)$ are the two design parameters in the design optimization, generating a leaflet area design space $\left( {160 mm^{2} \le LA \le 350 mm^{2} } \right)$.

The construction of the valve follows a two-step finite-element simulation conducted using ANSYS Mechanical Static-structural analysis with the HYPERFOAM material model (Ansys Inc., Canonsburg, PA, USA). As illustrated in Fig. 2c, each tube was oriented such that the cut sides that form the leaflets faced the global center and supported by a construction plane, which was a virtual plane to help constrain tube displacement. During the first step, tubes were stretched along the direction parallel to the construction plane via displacement boundary conditions, followed by a uniform pressure to flatten the tube (1,000 Pa was determined appropriate for these tubes to yield functional leaflets). At this point, the tube material facing the global center contacted the materials supported by the construction plane. By bonding the contacting material below the suture line near the bottom of each tube (material below the red region in Fig. 2b), we mimicked the suturing process such that the red regions became leaflets. The second step was to “roll up” the flattened tubes around the Z axis of the global Cartesian coordinate until the sides of the tubes contacted each other, yielding a closed-ring shape as the constructed TEHV. To achieve this, we applied remote displacement boundary conditions to rotate the construction planes, which drove the rotation of the flattened tubes (see Supplementary material 1 for animation). During rotation, a no-separation contact was enforced between flattened tubes and construction planes to smooth the bending. A frictionless contact relation was created between tubes to ensure proper closure of the valve. The augmented Lagrange method was used to model the contact interactions in this study. This penalty-based approach applies a Lagrange multiplier to enhance convergence and reduce penetration while maintaining high accuracy at a reasonable computational cost. A no-contact relation was set between tubes and other construction planes such that planes were free to virtually cross other tubes. For the two-step construction simulation, tubes were modeled with 10-node quadratic tetrahedral elements (e.g. 255,351 nodes and 145,293 elements for D = 15 mm and H = 9.5 mm). The simulation applied an adaptive time-step with ${\Delta }t_{min} = 1 \times 10^{ - 4} s.$

### Valve closure simulation

The first stage of valve geometry optimization was based on a valve closure simulation, where a uniform back pressure was applied on the outflow surfaces of the leaflets and valve root to model the pressure gradient during diastole. First, the solid stress in the constructed valve was fully relaxed, a limiting treatment of the poro-viscoelastic nature of the tube material. To ensure a well-posed computational problem, we adopted a method wherein the two ends of the valve were completely fixed (no displacement) while the domain in between the ends had no constraints to deformation or displacement, as indicated in Fig. 2a. To simulate valve closure and evaluate its performance, we applied pressure normal to the outflow surfaces of the leaflets and root. To enhance convergence, we utilized a linear ramp of pressure (Fig. 2d) increasing to 1,000 Pa (7.5 mmHg) over a duration of 1 s and holding for 0.5 s using an adaptive time-step with ${\Delta }t_{min} = 1 \times 10^{ - 4} s$ again. This pressure results in a diastolic pressure gradient approximately the pulmonary valve’s peak gradient reported in vivo (Silverthorn et al. 2019). We conducted a mesh resolution study and confirmed that increasing the number of elements by 50% does not result in qualitative changes in leaflet dynamics, ensuring the simulation is mesh-independent. A representative valve closure animation is seen in Supplementary material 2.

### Optimization of closed valve design

In this study, 26 design points based on the two design variables, tube diameter ($D$) and leaflet height ($H$), were considered for valve construction and closure simulation. To evaluate the valve performance (i.e. diastolic geometry), four measurable variables were used as design metrics. Their values were averaged over at least 20 equi-spaced timepoints during the 0.5 s time interval after reaching the peak pressure gradient (t = 1–1.5 s), which matches the diastolic period during a cardiac cycle. The four metrics are described below:

Coaptation area (CA) measures the total contact area between the three leaflets, determined by the ANSYS contacting algorithm to sum the area of elements with sliding or sticking contact status. The design with a larger CA during closure is favorable, as larger CA generally reduces valve regurgitation and prolapse propensity.

Regurgitation area (RA) is the total area of gaps between leaflets measured from the 2D short axis projection of a closed valve, as an estimate of the 3D leakage area. A smaller RA reduces valve leakage, and the ideal value is zero. The area was measured using the ImageJ software (NIH, Bethesda, USA).

Pinwheel index (PI) measures the degree of pinwheeling, which describes twisting of the leaflets, defined as:

$$PI = \frac{{l_{actual} - l_{ideal} }}{{l_{ideal} }}.$$

This parameter is commonly used to measure leaflet twisting in experiments (Midha et al. 2016) by comparing two quantities: $l_{actual}$ as the total length of all three free edges projected onto a 2D plane, and $l_{ideal}$ as the total free edge length of an ideally closed valve with straight coaptation lines. PI = 0 is the ideal value. A larger PI implies greater twisting of the leaflets. Pinwheeling is considered unfavorable as it can lead to fatigue damage and potential tearing of the leaflet. If PI < 0, the projected free edge is too short to allow for coaptation.

Prolapse area (PA) describes the degree of leaflet superposition by calculating the overlapping area between three leaflets based on the 2D projection of leaflet geometries. Prolapse indicates improper valve closure and increases the likelihood of valve leakage as fluid may pass through the gaps between overlapped leaflets. PA = 0 is the ideal value.

The closed valve design optimization was formulated and solved using ANSYS DesignXploer module, combining response surface optimization (RSO) with a multi-objective genetic algorithm (MOGA) to systematically explore the design space and locate an optimal solution (ANSYS Inc 2022a). The optimization problem was defined as minimizing a set of four objective functions: $f = \left[ {f_{1} \left( {\varvec{x}} \right), f_{2} \left( {\varvec{x}} \right),f_{3} \left( {\varvec{x}} \right), f_{4} \left( {\varvec{x}} \right)} \right],$ where each $f_{i} \left( {\varvec{x}} \right)$ corresponds to a performance metric listed above and ${\varvec{x}} = \left( {D,H} \right)$ is a vector of two key design variables. Specifically, we have four objectives: maximize CA $\left( {f_{1} \left( {\varvec{x}} \right)} \right)$, minimize RA $\left( {f_{2} \left( {\varvec{x}} \right)} \right)$, minimize PI $\left( {f_{3} \left( {\varvec{x}} \right)} \right)$, and minimize PA $\left( {f_{4} \left( {\varvec{x}} \right)} \right)$. Ideally, we may find an optimal solution that maximizes/minimizes all objectives. However, the more common situation is that conflicts exist between objectives. (e.g., a valve with more coaptation is likely to experience more pinwheeling.) Therefore, the problem needs to be solved by weighing each objective to minimize: $f = - \omega_{1} f_{1} \left( {\varvec{x}} \right) + \omega_{2} f_{2} \left( {\varvec{x}} \right) + \omega_{3} f_{3} \left( {\varvec{x}} \right) + \omega_{4} f_{4} \left( {\varvec{x}} \right),$ where $\omega_{i}$ are the weights for objectives summed up to 1 $\left( {\sum \omega_{i} = 1} \right)$ and the negative sign for $f_{1} \left( {\varvec{x}} \right)$ indicates the objective function needs to be maximized. In this study, we considered an equal contribution $\left( {\omega_{i} = 0.25} \right)$ of each listed metric. The influence of weight is discussed in a later section. Additionally, two objectives are constrained based on the metric definition: PI had to be non-negative (ensuring a fully closed valve, e.g. $PI \ge 0$), and PA, representing the overlapped area between leaflets, was also constrained to be non-negative (e.g. $PA \ge 0$).

The detailed optimization workflow is structured as follows: we first applied a face-centered central composite design (CCD) to establish a design of experiments (DOE) to explore the influence of ${\text{D}}\left( {15.625{\text{ mm}} - 16.875{\text{ mm}}} \right)$ and ${\text{H}}\left( {8.25{\text{ mm}} - 13.25{\text{ mm}}} \right)$ on the valve closing performance. To enhance resolution in the regions where the response surfaces exhibit high sensitivity to design parameters, D and H, additional design points were incorporated to result in the final 26 design points. Next, the 26 design points were constructed, and the valve closure simulations were performed. The time-averaged metrics were measured and were processed as a basis to yield four response surfaces via a genetic aggregation model, which uses a genetic algorithm to iteratively generate populations to screen and find the optimal response level for both D and H. Finally, MOGA was employed to find a set of Pareto optimal solutions. The method converged to the global Pareto solution by iteratively generating populations and applying selection, mutation, and crossover techniques until convergence was achieved. The final optimization yielded three potential Pareto sets for further screening, with detailed parameter selection and results discussed in a later section.

### FSI steady-flow simulation

The second stage of optimization selected three representative designs for subsequent FSI simulations to assess the sensitivity of flow-induced valve opening behavior to the design parameters D and H. One of these designs was near the optimal point identified in the first stage. The goal was to evaluate whether the optimal design determined during the diastolic closure phase also performs favorably during the systolic opening phase. A fully coupled two-way FSI simulation was performed using Ansys System Coupling, which coordinates two-way data transfers between parallel execution of two solvers: ANSYS Mechanical Static-structural analysis for the valve’s motion and ANSYS Fluent solver for modeling the hemodynamics. A fluid–solid interface was defined as the inner side of the tri-tube valve and shared by the two solvers for data transferring, where the deformed valve displacement was transferred from mechanical solver to fluid solver and fluid-induced wall forces from fluid to solid. The transfer repeated until the solutions converged between two solvers at the FSI interface. To account for the temporal change of the fluid domain due to the motion of the solid domain, a spring-based smoothing methodology was implemented to remesh the fluid domain to facilitate convergence at each timestep.

In the solid solver, the AHYPER material model was employed with the same boundary conditions as prescribed for valve closure. In the fluid solver, blood was modeled as an incompressible Newtonian fluid with density $\rho = 1005 kg/m^{3}$ and viscosity $\eta = 0.0041 Pa \cdot s$. The flow was assumed to be three-dimensional and laminar because we are primarily interested in the flow behind the leaflets, where fluid velocity is much less than the orifice jet and any turbulence effects are presumably negligible.

For boundary conditions, uniform fluid velocity was prescribed at the inlet and the velocity waveform used is presented in Fig. 2e. To facilitate convergence, the inlet velocity was gradually increased using a ramp until it reached the target velocity of 0.22 m/s, corresponding to a volumetric flow rate of 5 L/min. This flow rate approximates the typical time-averaged cardiac output of an adult at rest. Zero-gauge pressure was enforced at the outlet. For the fluid–solid interface, typical boundary conditions of no-slip and continuity of tangential and normal stresses were imposed. The fluid motion governed by the incompressible Navier–Stokes and continuity equations is solved by a double-precision pressure-based transient solver in ANSYS Fluent based on the finite volume method. The pressure and velocity are stored using a cell-centered approach and are updated sequentially using a semi-implicit method for pressure linked equations (SIMPLE) algorithm. For spatial discretization, the pressure is discretized with the second order scheme and the gradients with the least squares cell-based method. The momentum is discretized with a second-order upwind scheme.

The solid and fluid domains were discretized with 10-node quadratic tetrahedral elements (e.g. 402,803 nodes and 234,092 elements for solid, and 3,183,319 nodes and 2,266,693 elements for fluid in Case A, D = 15 mm, H = 12 mm in Fig. 5). The system coupling for FSI used a fixed time step with ${\Delta }t = 1 \times 10^{ - 3} s$, with 5 iterations for communication between mechanical and fluid solvers to ensure convergence at the FSI interface.

## Results

### Mechanical testing

**Fig. 3:**
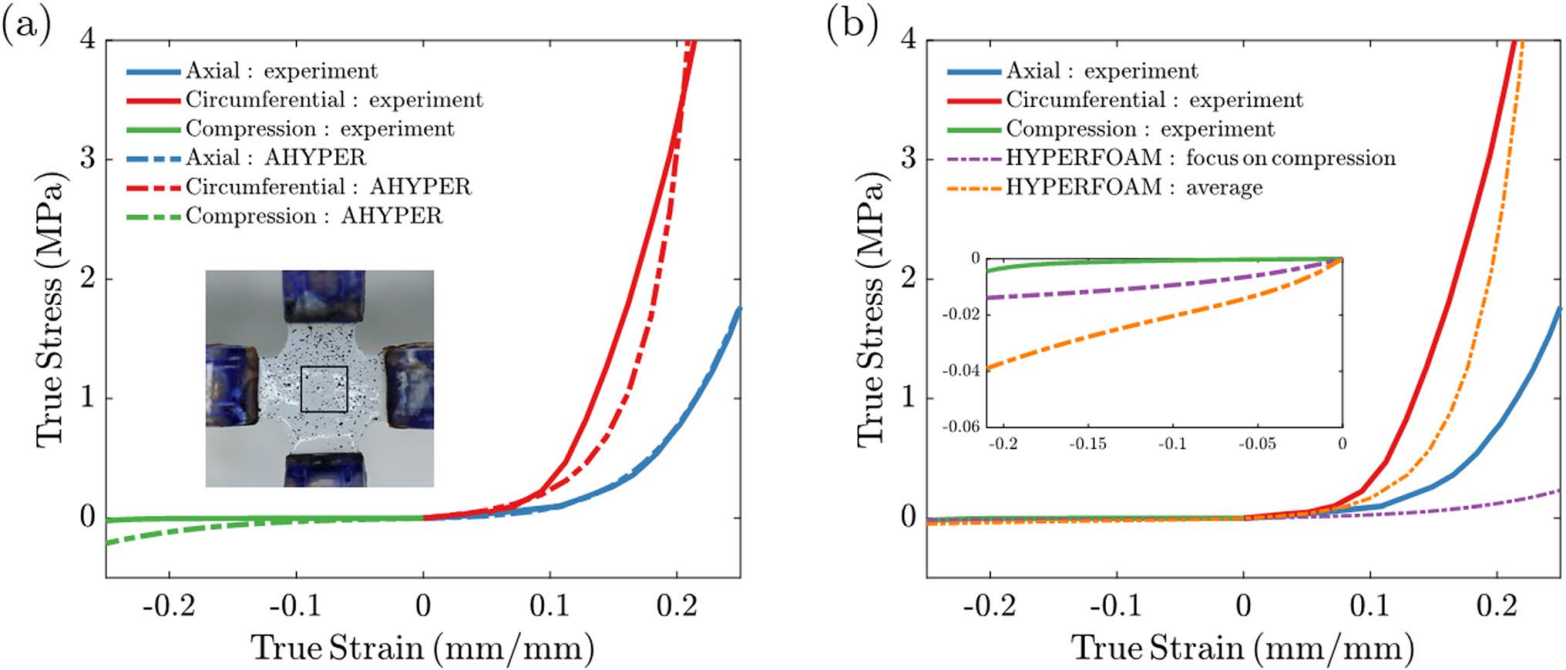
Tube stress–strain behavior and constitutive model fits: **a** AHYPER model in axial and circumferential directions by fitting high strain rate tension data from planar equi-biaixal testing and low strain rate unconfined compression data using Mclibration. Inset: example of a test sample with speckles placed on the cruciform for strain tracking. The gauge area is demarcated by the black square. **b** HYPERFORM model used for improved fitting of unconfined compression data and used in valve construction simulation. Two fitted curves shown: the purple fit focused on compression data and the orange fit included averaged tension data. Inset: zoomed fit in compression

Planar equibiaxial tension and unconfined compression tests were performed on biologically-engineered tube samples. Detailed equibiaxial testing results can be found in S10 (Supplementary). The averaged stress–strain data are presented as solid lines in Fig. 3. The dominant stress along the circumferential direction compared to the axial direction confirms the mechanical anisotropy of the tube, consistent with circumferentially aligned collagen fibers previously reported (Syedain et al. 2016, 2017).

The high strain rate (~ 75%/s) planar equibiaxial data were used to fit the AHYPER model in Eq. 1, which was used in the valve closure and FSI simulations where leaflet loading at contact occurs at high strain rates. We obtained material constants $a_{1} = 0.0001 MPa$, $a_{2} = 0.2 MPa$, and $a_{3} = 0.34 MPa$ for isotropic contribution, fiber strength $c_{1} = 0.44 MPa$ and $c_{2} = 7.6$ for the circumferential direction, and $e_{1} = 0.000001 MPa$ and $e_{2} = 3.5$ for the axial direction, and incompressibility parameter $d = 0.002 MPa^{ - 1}$, which is obtained from the bulk modulus of the descending thoracic aorta (Carew et al. 1968). Though the incompressibility assumption implies $d = 0$, we have verified that the deviation in $d$ results in less than 5% error in stress in uniaxial tension simulation, which justifies the assumption of incompressibility in calculating the true stress from W for comparison to the measured engineering stress that was converted to true stress based on that assumption. As expected, the two fiber directions sufficiently captured the known circumferential collagen fibril alignment in the tubes. The prefactor was dominant in one direction $\left( {{\text{c}}_{1} /{\text{c}}_{2} { } \gg {\text{e}}_{1} /{\text{e}}_{2} } \right)$, while the other direction showed negligible contribution $\left( {{\text{e}}_{1} /{\text{e}}_{2} { } \approx { }0} \right)$. The AHYPER fit in Fig. 3a captured the anisotropic tissue behavior and predicted axial tension data with high accuracy. Along the circumferential direction, AHYPER underpredicted stress for strains between 10% and 20% and overpredicted stress for higher strains. 

The AHYPER model shows a poor fit for compression data for greater (more negative) compression. The unconfined compression test was performed under a low strain rate. In addition, during valve suturing, material deformation is quasi-static where loads are applied relatively slowly. Specifically, compression of tissue arises from flattening (step 1) and bending (step 2) the tissue tubes. Therefore, the AHYPER model obtained from high strain rate data may not accurately describe the construction, especially the compression regime, where the AHYPER prediction deviates from the much softer material response for (true) strains < − 0.15. Consequently, we applied an isotropic HYPERFOAM model mentioned previously for the in silico valve construction procedure. This yielded a better fit of the compression regime. The first fit (orange curve in Fig. 3b) with material constants $\mu = 0.01 MPa$, $\alpha = 27.8$ and $\beta = 0.274$ was based on compression data and tension data averaged by two directions. The second fit (purple curve in Fig. 3b) with material constants $\mu = 0.01 MPa$, $\alpha = 14.5$ and $\beta = 0.0689$ focused on the compression data and was used for the results described below. A comparison of constructed valves resulting from the two HYPERFOAM models is included in Supplementary S11 and discussed below. The resulting underestimation of the high strain rate tensile stress is actually consistent with the stress relaxation properties of tissue in quasi-static tension (Robinson & Tranquillo 2009).

During construction simulation, the HYPERFOAM model effectively captured the stronger compression that resulted. We include strain distribution plots (S12, Supplementary) for the tube flattening step, where large deformation with high compressive strain (minimum principal strain) was observed along the cut edge and at the round corners of the flattened tube. The compressive strain was significantly greater in magnitude than the tensile strain, justifying the use of the HYPERFOAM model for the valve construction simulation. The largest compressive and tensile strain during construction occurred during the tube flattening step, where the peak tensile strain ($\approx 0.{15}$, as shown in Supplementary S12) remained within the fitted range, while the compressive strain ($\approx - 0.{46}$, also in Supplementary S12) exceeded the HYPERFOAM fitted range. However, since the peak strain was highly localized to the edge where material was resected from the tube, and the compressive strain elsewhere remained above $- 0.{2}$, we consider HYPERFOAM a valid model to construct the valve. The strain distributions for a snapshot during valve closure, where the AHYPER model was used, are shown in Supplementary S12. Peak tensile $\left( { \approx 0.{34}} \right)$ and compressive strain $\left( { \approx - 0.{27}} \right)$ regions were primarily localized around the commissures, while strains for the rest of the valve $\left( { \approx { } - 0.{15 } - \, 0.{19}} \right)$ remained within the fitted range. Compared to valve construction, valve closure results in higher tensile strain magnitudes and lower compressive strain magnitudes, justifying the use of AHYPER for the valve closure simulation.

### Validation of valve closure simulation

The computational model for valve closing simulation based on these constitutive models was first validated against experimental data previously reported for a 19 mm tri-tube valve constructed from 16 mm tubes (Syedain et al. 2021). The valve was constructed using the two-step framework proposed herein with D = 13.5 mm and H = 11 mm, a slightly smaller tube size being used to account for tissue folding during suturing of the tubes together that effectively decreases the tube diameter.

**Fig. 4:**
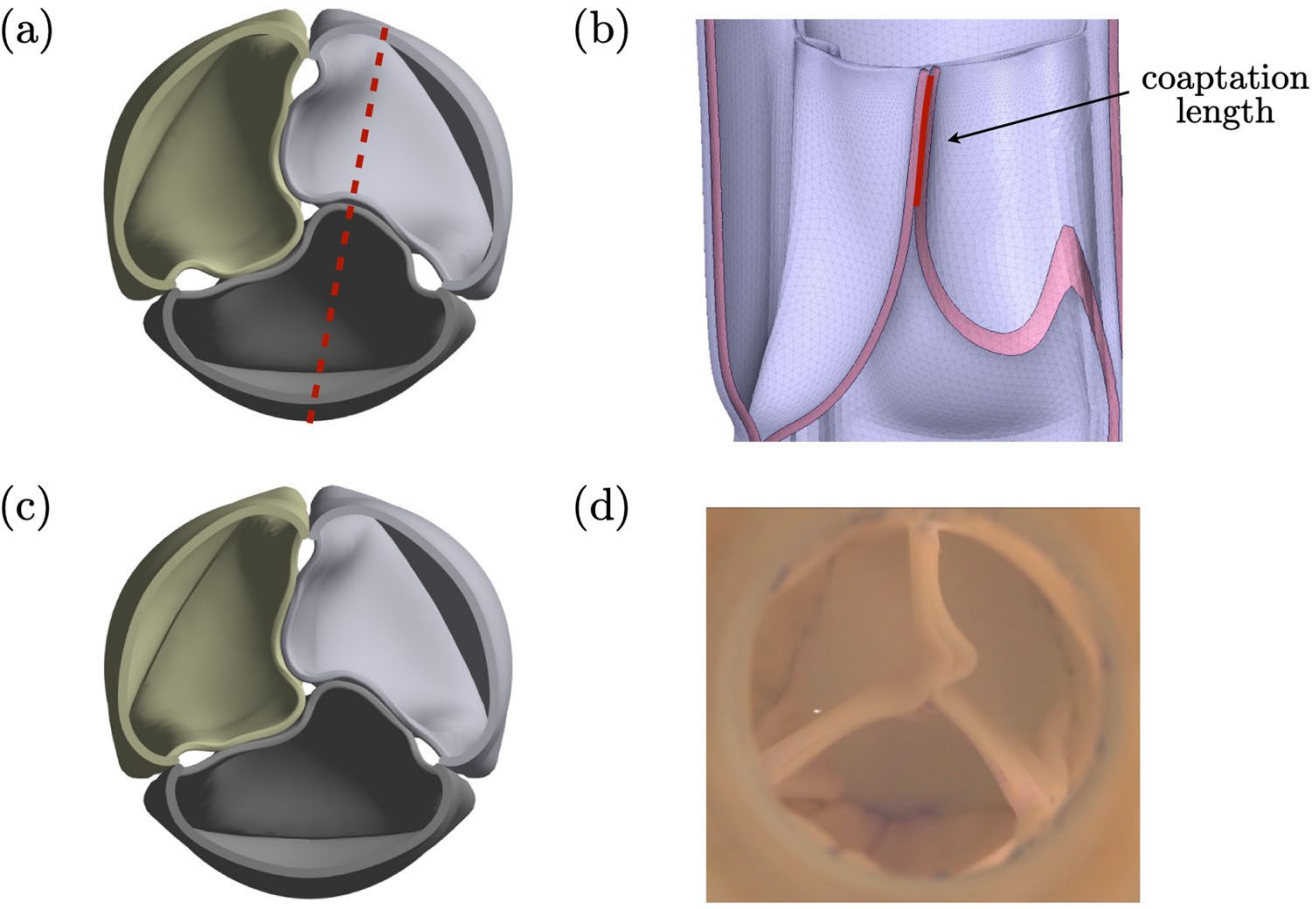
**a** Simulation snapshot of a closed valve under 7.5 mmHg. The dashed line represents the plane defined to measure coaptation length. **b** The plane view illustrates the measurement of coaptation length, which is defined as the red line. **c** Simulation snapshot of a closed valve under 16 mmHg. **d** Pulse duplicator snapshot of the closed valve

Two key experimental observations were used to validate the model. The first was the coaptation length measured in vivo from long axis echocardiographic images of an implanted valve in a sheep, which showed a coaptation length of 5 mm in peak diastole (Syedain et al. 2021). We performed a valve closure simulation using the reported peak pulmonary diastolic gradient in sheep (7.5 mmHg) (Silverthorn et al. 2019) and extracted the coaptation lengths for the three leaflet contacts in the simulation. These values of 5.6 mm, 5.2 mm, and 5.5 mm compare well to the experimental value of 5 mm. Simulation snapshots illustrating the coaptation length extraction are shown in Fig. 4b.

The second validation compared the closed valve geometry observed in the pulse duplicator under cyclic loading, where the averaged diastolic pressure gradient was estimated as 16 mmHg from Supplementary Figure S3A in (Syedain et al. 2021). Though the experimental short axis snapshot was taken from a pulsatile waveform, the valve shape remained stable during the diastolic phase in experiments, allowing comparison to the simulation. In the valve closure simulation using a 16 mmHg gradient, the closed valve exhibited a similar pinwheeling pattern for one leaflet and also root deformation (as seen along the lateral suture line at the bottom of each tube) to that in Fig. 4. Due to the slight asymmetry of the experimental valve leaflets, the simulation did not fully capture the observed free edge pattern. Nevertheless, the reasonable agreement in coaptation length and closed valve shape between simulation and experiment validates the current model as a useful tool for the first-stage design screening.

### Valve closure simulation and geometry optimization

**Fig. 5:**
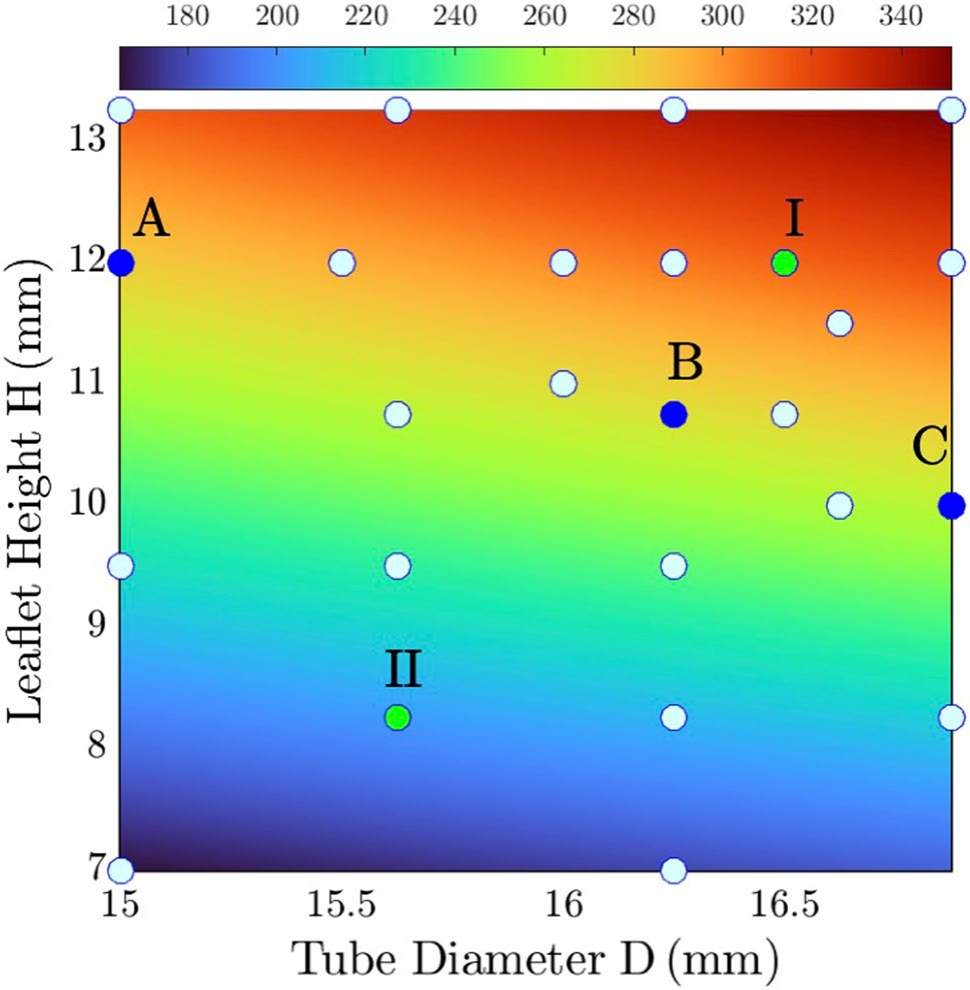
Design space of leaflet area. Solid circles mark 26 design points. Green markers represent two examples (Cases I and II) discussed subsequently. Blue markers show three design points selected (Cases A-C) for FSI simulation

We performed the two-step construction on 26 design points spanning the design space of the leaflet area shown in Fig. 5. The valve construction workflow yields unique valve shapes determined solely by the design parameters, D and H, and a larger leaflet area can be obtained with increased D and/or H.

**Fig. 6:**
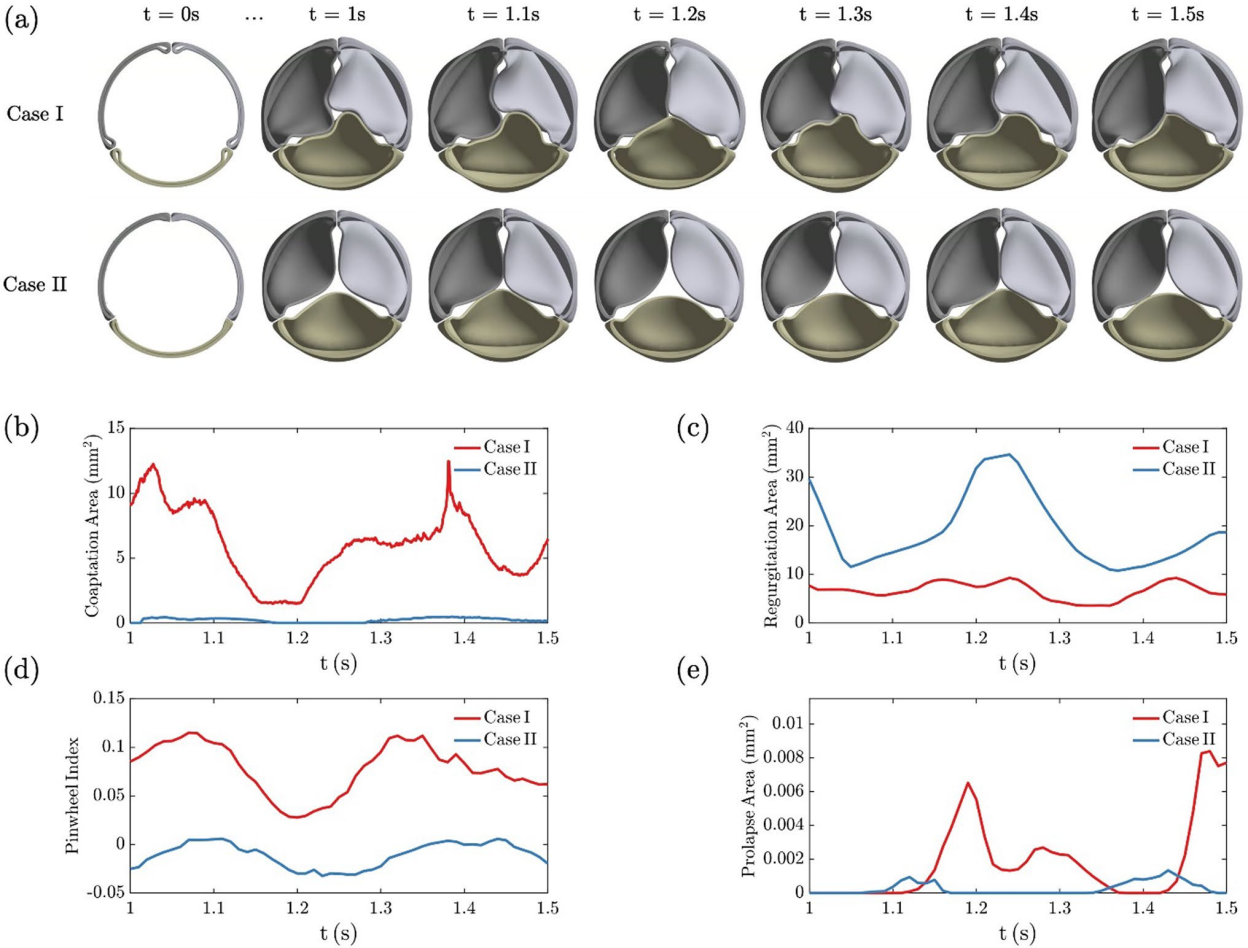
Comparison of two design points for dynamics from 1 -1.5 s during valve closure simulation. Case I (D = 16.5 mm, H = 12 mm, A = $311 m{m}^{2}$) and Case II (D = 15.625 mm, H = 8.25 mm, A = $202 m{m}^{2}$). **a** Top view of leaflet shape evolution. Measured metrics during t = 1–1.5 s: **b** coaptation area, **c** regurgitation area, **d** pinwheel index, and **e** prolapse area. See animation in Supplementary material 3 for the evolution of these two cases

The performance of all design points during valve closure simulation was evaluated based on the four metrics stated in 2.4. To address the influence of leaflet area, we selected two design points for comparison (green markers in Fig. 5, Cases I and II), where Case I has a larger leaflet area ($311 m{m}^{2}$) than Case II ($202 m{m}^{2}$). Figure 6a shows the temporal evolution of leaflet shape within the 0.5 s interval considered for optimization and the measured metrics are presented in Fig. 6b-e.

From snapshots in Fig. 6a, valves were fully opened at t = 0 s resulting from construction. The ramped diastolic pressure gradient pushed the leaflets inward to close the valve. At t = 1 s, relatively closed states were achieved and sustained after reaching the target pressure gradient. Throughout the 0.5 s time interval considered for optimization, Case I with a larger leaflet area demonstrated stronger interactions between leaflets with more twisted contact lines. Case II showed less leaflet interaction with relatively straight coaptation lines, but also a central gap.

The evolution of the coaptation area in Fig. 6b records fluctuations due to strong elastic interactions between leaflets. The transient evolution of pinwheeling in Fig. 6d tracks coaptation. For Case I, both figures indicate two coaptation area peaks around t = 1 s and t = 1.4 s, where leaflets are in a twisted conformation that increases the contact in the leaflet belly. The minimum coaptation at t = 1.2 s leads to symmetric coaptation lines (therefore small pinwheeling) where leaflets slightly contact each other. Prolapse is evident for the left leaflet at t = 1.2 s and the right leaflet at t = 1.5 s, matching two peaks in Fig. 6e.

Though small gaps were captured around the commissure, Case I shows a small regurgitation area in Fig. 6b. In contrast, Case II reveals poor coaptation and a persistent regurgitation gap is also observed at the center. Despite this, Case II demonstrates almost straight coaptation between leaflets without prolapse. Without enough leaflet area, leaflets show slight contact lines, and the valve fails to remain closed, leading to a peak around t = 1.2 s in the regurgitation area in Fig. 6c. This fact is also reflected in pinwheeling as the index becomes negative for the leaked valve. Based on comparison, the design with a larger leaflet area shows better coaptation, less regurgitation, more pinwheeling, and more prolapse. 

**Fig. 7:**
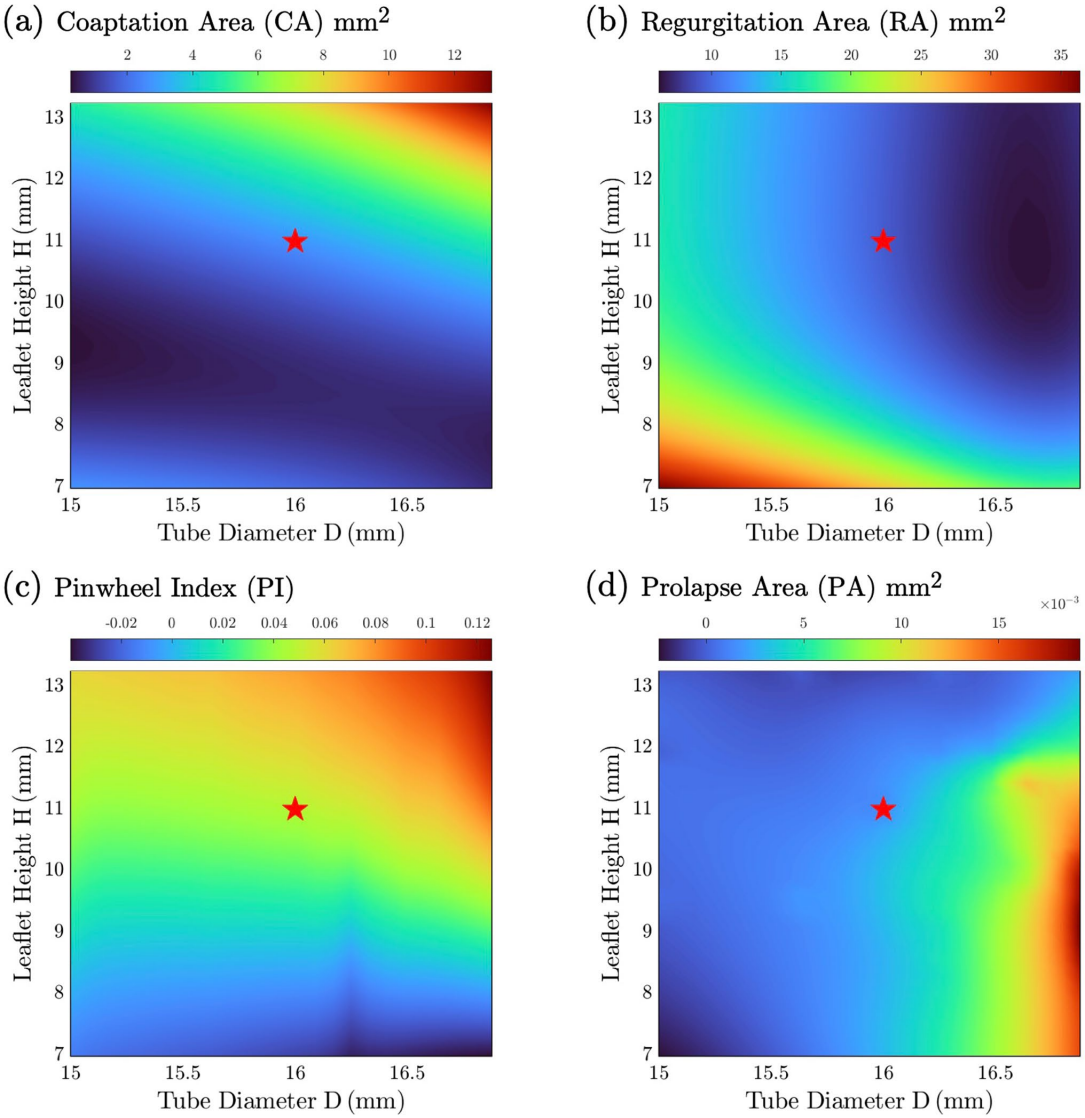
Response surfaces of metrics: **a** coaptation area, **b** regurgitation area, **c** pinwheel index, and **d** prolapse area. The star labels the optimal design resulting from optimization

The two selected cases illustrate the influence of the leaflet area on the four design metrics, providing insights into the responsive surfaces generated from the 26 design points. Figure 7 presents the response surfaces for each metric, where the plots provide a direct response of measured quantities based on changes in tube diameter D and leaflet height H (thereby the change in leaflet area in Fig. 5).

In Fig. 7a, the coaptation area increases with the leaflet area, and the maximum coaptation is achieved at the top right corner of the response surface with the largest tube diameter D and leaflet height H (D = 16.875 mm, H = 13.25 mm). The regurgitation response surface (Fig. 7b) is almost the mirror image and indicates a smaller regurgitation can be obtained with a larger leaflet area, whereas the design with minimal regurgitation occurs with the largest D but slightly smaller H (D = 16.875 mm, H = 9.9 mm). Like coaptation area, pinwheeling (Fig. 7c) also increases monotonically with larger D and H, but with a different gradient. In contrast, the response surface for prolapse (Fig. 7d) has a more complicated topology. It is greater for designs with larger D and small or intermediate H but smaller for designs with larger H. It shows some regions with a slightly negative prolapse area arising from interpolation between known design points, and so these design points were excluded. Consistent with the observation made from the design points comparison, the response surfaces demonstrated a direct influence of leaflet area, determined by both D and H, on the metrics, including coaptation area, regurgitation area, and pinwheeling. On the other hand, prolapse exhibited greater dependence on D when H is small, while this effect is less pronounced as H increases.

The optimization of valve geometry was performed using MOGA with equal weight assigned to the four design metrics. In addition, target values were specified during optimization to offer the solver an estimated and achievable goal value for each metric, as required by MOGA (ANSYS Inc 2022a). We applied target values as CA = 3 $m{m}^{2}$, RA = 0, PI = 0 and PA = 0. The target value for CA was an intermediate value among all existing designs. The rest of the target values were the ideal values for each metric. Unlike constraints, the target value is not strictly enforced and will be replaced if a better solution can be found. The method initially generated 2000 samples and assigned 400 samples per iteration with 20 iterations to obtain candidate points. The maximum allowable percentage of Pareto was set at 70%, meaning that the optimization terminates when the resulting front of solutions contains at least 70% of the samples per iteration (280 in this case). The conditional stability was set at 2% and the optimization converged after 3755 evaluations. An optimal design point was identified (D = 16 mm and H = 11 mm), marked as the star in Fig. 7, which is a global optimum by visual inspection of these response surfaces. An animation of valve closure of the optimal valve design is shown in Supplementary material 2. This design point predicts the optimal 24 mm tri-tube valve performance in diastolic closure in terms of maximizing coaptation and minimizing regurgitation, pinwheeling, and prolapse.

### Valve opening FSI simulation

After identifying the optimal design from diastolic closure simulation under constant back pressure, we probed the systolic hemodynamics by performing a two-way FSI simulation. The flow waveform in Fig. 2e was used. Three selected designs with similar leaflet areas, labeled as blue markers in design space (Cases A-C) in Fig. 5, but with varied tube diameter D and leaflet height H were assessed. The initial valve geometries were selected from the valve closure simulations to be of similar geometric orifice area (GOA), and taken to be stress-free states. Cases A and C are located on the boundary of the design space, with large H and D, respectively. Case B is situated near the optimal design from the optimization with intermediate D and H.

**Fig. 8:**
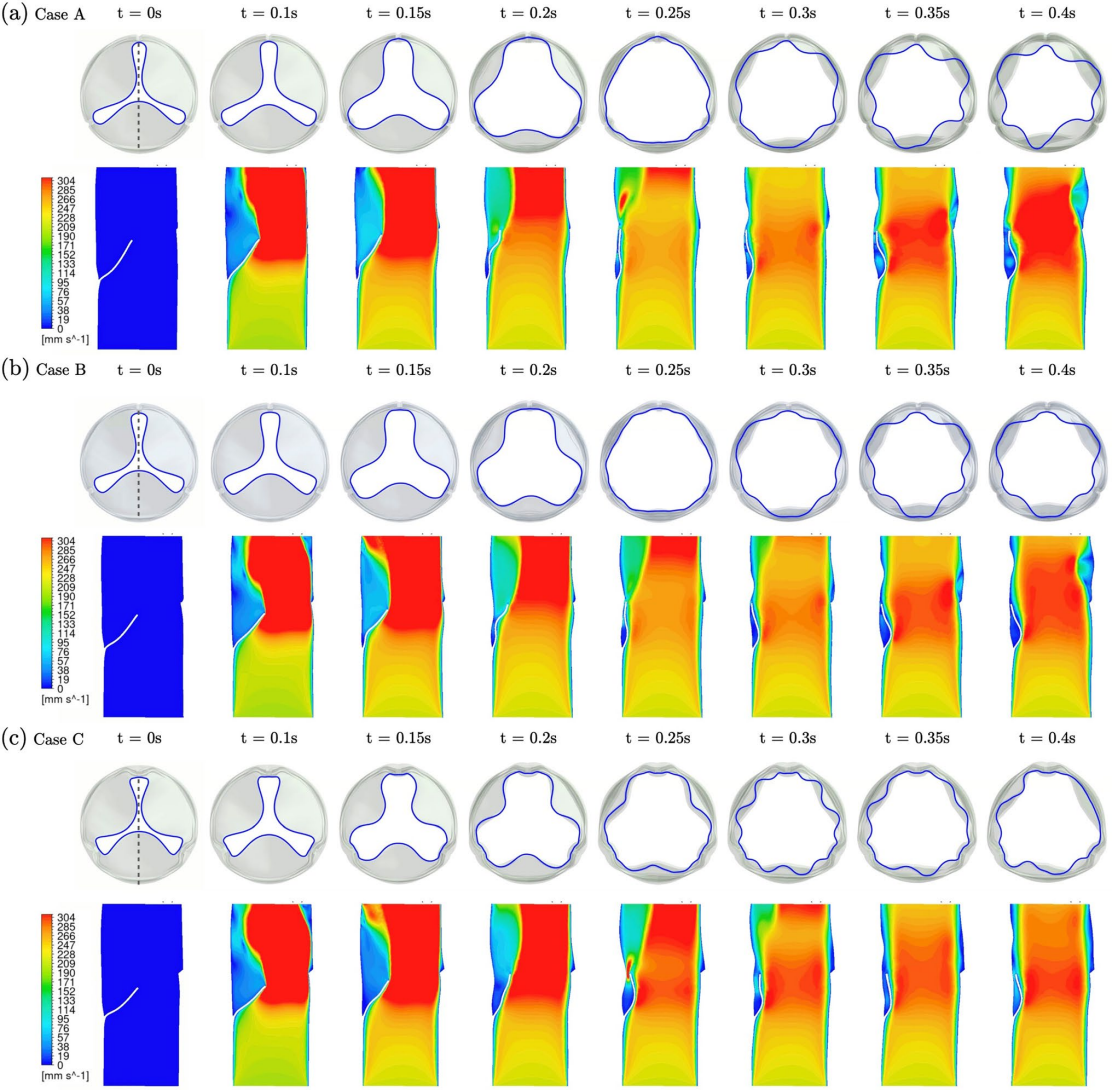
Evolution of the free edge (top view) and velocity contour slice (side view) for three selected designs of approximately the same leaflet area and GOA. The dotted line at t = 0s indicates the location of the slice view. **a** Case A: D = 15 mm, H = 12 mm. **b** Case B: D = 16.25 mm, H = 10.75 mm. **c** Case C: D = 16.875 mm, H = 10 mm. See animation in Supplementary material 4 for the evolution of these three cases

Figure 8 shows the evolution of the free edge and a slice of the blood velocity contour focused on the period t = 0.1 s–0.4 s, with a snapshot indicating the initial state at t = 0 s. The top panel, as a top view, tracks the leaflet free edge deformation. The bottom panel presents a slice view of the axial velocity, following the dotted line shown at the top panel when t = 0 s. From the top panel, all three valves have similar initial GOA, with no flow at t = 0 s. When the inflow reaches v = 0.22 m/s at t = 0.1 s, the valves begin to open. The GOA keeps increasing until all three cases achieve a fully opened state at t $\approx$ 0.25 s where the maximum GOA is achieved. This is followed by oscillation of the free edge in all cases, where the oscillation is more pronounced with increasing tube diameter: Case C with the largest diameter (D = 16.875 mm, thus more free edge) shows a stronger oscillation frequency between t = 0.25 s – 0.4 s compared to Cases A and B, which also exhibit smoother, less scalloped edges.

From the velocity contour panel, leaflets (indicated as white lines) are initially concave in all cases. The leaflets become deformed due to inflow and develop into a convex shape at t $\approx$ 0.15 s. The deformation traverses along the leaflet toward the free edge. When the valve becomes fully opened at t $\approx$ 0.25 s, the leaflets in all cases revert to the concave shape as the large GOA is attained. Here, the variation in H results in different extents of close contact between the leaflets and the wall: in Cases A and B, the top half of leaflets experience close contact. Case C, with a smaller leaflet height, results in only a small contact region near the free edge, which induces a strong local jet near the contact point. In the later stage of simulation (t = 0.25 s–0.4 s), the hemodynamic interaction between leaflets and blood flow as well as the deformable valve root may explain the break in the initial spatial symmetry.

**Fig. 9:**
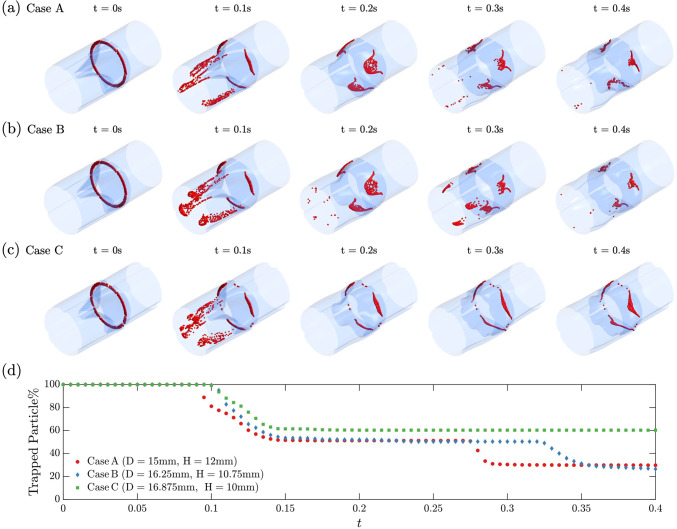
Particle tracking snapshots for three selected designs. **a** Case A: D = 15 mm, H = 12 mm. **b** Case B: D = 16.25 mm, H = 10.75 mm. **c** Case C: D = 16.875 mm, H = 10 mm. **d** Evolution of particles “trapped” (retained) in the simulation domain. See animation in Supplementary material 5 for the evolution of these three cases

Besides the leaflet shape and motion dynamics, we probed the hemodynamics behind the leaflets in response to the complex motions of the leaflets during systole with a particle tracking analysis. A ring of 2000 massless particles was initially distributed close to the base of the leaflet in each design (t = 0 s in Fig. 9a-c), such that most of the particles were initially behind the leaflets. The particle motions and residence times were tracked. Figure 9a-c shows the temporal evolution of particle tracking snapshots, corresponding to snapshots in Fig. 8. The percentage of the particles remaining in the simulation domain is presented in Fig. 9d, where two drops were observed. The first drop at t $\approx$ 0.1 s is pronounced in all three cases. It is caused by particles initially located on the inlet side of the leaflets that are carried away by the translating flow and leaving the domain. By t = 0.2 s, nearly all particles from the first drop leave the simulation domain. Those particles are quickly carried by the jet and are not the focus of our study. The deviation in the remaining particle percentage is due to the different leaflet configurations in the three valve designs. The second drop at t $\approx$ 0.28 s represents the particles initially behind leaflets leaving the domain. Interestingly, no particles escaped in Case C, while many, but not all, of the particles escaped in Cases A and B, and this trend is sustained until the end of the simulation (t = 0.4 s).

## Discussion

In mechanical testing, a cruciform sample clamped with grips was stretched equibiaxially under a high strain rate consistent with leaflet contact upon coaptation. The cruciform shape focuses the applied load in the central region. However, the clamping method introduces stress concentration along the cruciform corners leading to stress shielding of the central region where strain is tracked (Jacobs et al. 2013; Sun et al. 2005). Studies have shown that a tether-based method like hooks alleviates the boundary effects (Sun et al. 2005), but this may introduce tissue damage and fixation instability during preconditioning cycles. Different material constitutive models were employed in this study to simulate valve construction and closure. The HYPERFOAM model, albeit an isotropic model, better captured the material’s compression behavior during the quasi-static two-step construction simulation, where compression dominated over tension. This was confirmed by strain distributions for a specific design point (D = 16 mm, H = 11 mm) during the tube flattening step of valve construction shown in Supplementary S12. The results showed that compressive strain was of greater magnitude than tensile strain. On the other hand, the AHYPER model accounted for the material’s anisotropy under tension, making it more suitable for valve closure where forces within coapting leaflets are biaxial. The strain distribution plot from a representative snapshot during closure simulation revealed that most leaflet regions experienced tensile strain < 0.2 and compression strain > − 0.1, both within the range of fitted data from mechanical testing (Fig. 3). Only a minimal portion of elements (less than 0.1%) around the commissure exhibited high tensile and compressive strains beyond the testing range.

In the first stage of design optimization of the tri-tube valve, an optimal design (D = 16 mm, H = 11 mm, marked as a star in Fig. 7) was obtained based on the performance of designs during a valve closure simulation due to an imposed back-pressure. This approach, saving the computational costs of performing full two-way FSI simulations for every design point, achieves an efficient and robust design screening based on diastolic valve geometry. Response surfaces revealed relations between the design variables (tube D and H) and performance metrics, and they enabled the optimization. It is worth noting that the accuracy of the interpolated response surfaces depends on the quantity and quality of the input data, and they may not accurately capture the behavior of the valve for all designs. The current study employed 26 design points and provided reasonable coverage of the design space especially in regions most sensitive to D and H.

Under a diastolic pressure gradient, the tri-tube valve closed from an initially unloaded fully open state, involving complex leaflet interactions. Significant inward bending of the leaflets and resulting coaptation were observed, along with noticeable inward deformation of the root near the base of the leaflets (see animation in Supplementary material 2). However, the closure of the valve was not perfect. In Fig. 6a, both cases have a small gap near the commissure. This gap becomes more pronounced with increasing tube diameter, as a larger tube requires more curvature near the commissure to accommodate the target 24 mm diameter valve. However, this observation is consistent across all design points. The likely explanation is the limited range of accuracy in the fitted AHYPER model used in the valve closing simulation. Specifically, in Fig. 3a, the AHYPER fit overestimates compressive stress for strains < − 0.1, leading to artificial stiffening. The magnitude of compressive strain in the commissure region during valve closing is substantially larger than 0.1 (Supplementary S12b). The tensile stress around the commissure (~ 0.34) also exceeds the fitting range, as indicated in Fig. 3a, which overpredicted the stress and contributed to the stiffening. As a result, the "stiffened" commissures failed to overcome the curvature from the initial flattened tube geometry and coapt properly, leading to the observed gaps. To address this limitation, future work will focus on improving the material model to better capture the material’s stress–strain behavior.

The four metrics to evaluate diastolic performance were chosen to cover various aspects of valve geometry and function. Other metrics, such as the maximum equivalent stresses, could be considered to further improve optimization. We did not include this metric here as the maximum equivalent stresses in the simulation were lower than the ultimate tensile stress (UTS), which is around 3 MPa for the biologically-engineered matrix (Syedain et al. 2021). For instance, the maximum equivalent stress for the optimal design during the considered closing period of 0.5 s is 1.97 MPa, occurring at the commissure, agreeing with previous studies (Hsu et al. 2015; Lee et al. 2020, 2021).

Pinwheeling was observed in some valve closure simulations, especially pronounced in the design points with larger tube diameters (see Fig. 7c). The uniform leaflet shape became unstable and started to twist around the central axis. This leaflet twisting could potentially lead to tissue fatigue. Prolapse, measured as the overlapping area due to leaflet superposition, occurred at the free edge when sufficient leaflet area existed to allow overlapping. Therefore, prolapse occurred more frequently for designs with more free edge length (i.e. greater D), rather than simply greater leaflet area. This outcome is evident in Fig. 7d, where the prolapse area increases monotonically with D. Interestingly, the prolapse area remains small for design with large H (H > 12 mm), even when D is large. One potential explanation is that more leaflet height increases leaflet area without introducing more free edge length, allowing more coaptation as a support for stable coaptation between leaflets. 

The optimal design (D = 16 mm, H = 11 mm) for diastolic closure geometry resulted from equal weights for all four design metrics. By adjusting the weights and targets of specific metrics used in MOGA, alternative optimal designs can be tailored to meet different performance priorities. For example, when considering the growth potential of a smaller tri-tube for pediatric use, the optimal design should have an excess coaptation area for the grown valve to remain functional as the leaflets grow. When greater importance is assigned to the coaptation area, the resulting optimal point shifts toward a larger leaflet area (i.e. increased D and H).

In the second stage of optimization, steady flow simulations with particle tracking were performed for three selected design points with similar leaflet areas and initial GOA for hemodynamic comparison. The observed fluttering of leaflets has been reported in many experimental and computational studies. Fluttering may lead to leaflet tissue fatigue and valve failure and is closely related to valve design and leaflet geometry. Lee and coworkers proved in experiments with computations that valves with smaller diameters or thicker leaflets show more severe fluttering (Lee et al. 2021). Interestingly, a recent computational study shows that thinner leaflets induce more fluttering (Johnson et al. 2020). In this work, we probed the problem by examining designs with different free edge lengths. Three FSI cases share a similar leaflet area, valve size, and leaflet thickness, but different D and H. In Fig. 8, the difference in design parameters leads to qualitatively different responses to leaflet fluttering motions: a design with a larger tube diameter (more free edge) indicates more fluttering with a scalloped free edge shape. For instance, Case C with the smallest H demonstrates a more complex orifice at t = 0.3 s compared to the other two designs, due to the excess free edge.

The washout analysis based on tracking massless particles suggests that the flow circulation behind leaflets strongly depends on leaflet height H. As mentioned, the incoming flow pushes the leaflet toward the valve wall to open the valve and triggers a wavy deformation propagating from the root to the free edge. For designs with larger leaflet height (Case A and Case B), the leaflet deformation, along with the complex interactions with the valve wall, squeezes the fluid behind the leaflet (see t = 0.2 s in Fig. 8). Consequently, particles initially behind the leaflets successfully escape. Subsequently, the near contact between leaflets and wall (at t = 0.25 s) creates an upward stream that accelerates particle escape. In contrast, the leaflet in Case C fails to squeeze the fluid behind the leaflets due to its smaller height. Though the leaflet tip generates an upward stream at t $\approx$ 0.25 s, the deep region behind the leaflets is slightly altered, leading to poor washout of particles. This is where a sinus is located in a native valve, which contributes to effective washout (Motta et al. 2019), and could be the basis for future design improvement.

There are nuances regarding FSI of heart valves that deserve discussion. In the FSI setup, the initial valve geometry derives from a snapshot during valve closure simulation, in which the valve is slightly opened. The solid stress in the geometry developed during valve construction was relaxed before the FSI simulation, which can be considered a limit of the actual poro-viscoelastic nature of the engineered matrix that is not captured in the purely elastic AHYPER model. Relaxing stress in the solid structure before FSI was likewise done in previous studies (Sigüenza et al. 2018; Spühler et al. 2018). However, it remains an interesting question to consider the unloaded state of a valve in FSI simulation. For prosthetic valves, the choice of reference state depends on the leaflet material and fabrication process, especially for leaflets made of polymers. For example, Kamensky et al. (2017) modeled an artificial heart valve with a flat stress-free state for latex leaflets and implemented pre-strained and slightly opened initial configurations for FSI simulations. Sigüenza and coworkers (2018) applied a closed valve geometry to replicate the heat treatment of polymeric leaflets used in their experiments. They started with a fully opened valve and used normal pressure to close the valve. The resultant closed state was used as the initial geometry. A half-opened initial shape similar to this study was used by Spühler et al. (2018). For bioprosthetic valves, Oks et al. (2022) constructed a partially opened valve as the unloaded state. Kaiser et al. (2021, 2023) computed a loaded geometry that sustained diastolic pressure from a set of equilibrium equations and used it to further predict an opened valve as a pre-strained initial configuration. They examined the variation in pre-strain, thereby different reference states, and suggested that it has small effects on valve closure configuration. However, this conclusion likely depends on the degree of nonlinearity in stress–strain behavior.

In this work, we solely focused on simulating steady flow through the tri-tube valve rather than pulsatile flow. Although steady flow simulations provide insight into the hemodynamics of a valve design, a comprehensive investigation of the entire cardiac cycle necessitates the simulation of pulsatile flow to capture phenomena such as closing volume and true washout during valve closure. The arbitrary Lagrangian–Eulerian (ALE) method used for FSI in this study discretizes solid and fluid domains separately with geometrically conforming meshes, offering high accuracy in force and displacement at the fluid-sloid interface. However, convergence is an inherent technical issue because of large deformation in both fluid and solid domains. While the results are highly accurate, the limitation of efficient, guaranteed convergence with ALE makes FSI for even one valve cycle computationally demanding. The immersed boundary (IB) method treats the solid as a subdomain immersed in the fluid, allowing it to simplify the simulation domain without remeshing interface but at the expense of lower accuracy. For future work, the IB method will be implemented to characterize the tri-tube valve performance in pulsatile flow by simulating multiple cardiac cycles to ensure the conclusion from steady-flow washout FSI is unchanged and to characterize the wall shear stress over the cardiac cycle as it affects phenotype of endothelial cells that appear during regeneration.

## Conclusion

A two-stage framework to efficiently optimize the geometry of a novel tri-tube heart valve design comprising valve construction/closure simulation under steady back pressure to screen the full design space, followed by FSI simulation of steady flow to analyze the relevant subspace, is demonstrated. The combination of response surfaces and MOGA yields an optimal design based on a tube diameter *D* = 16 mm and leaflet height H = 11 mm to optimize competing objectives of maximizing coaptation area, and minimizing regurgitation area, pinwheeling, and prolapse at valve closure for a tri-tube valve constructed from biologically-engineered tubes of circumferentially-aligned collagenous matrix. The optimal design point yields superior washout under steady flow compared to other design points with the same leaflet area and smaller leaflet height. Simulation of pulsatile flow is still required for complete optimization.

## Supplementary Information

Below is the link to the electronic supplementary material.

Supplementary file1 (MOV 1914 KB)

Supplementary file2 (MOV 3714 KB)

Supplementary file3 (MOV 1855 KB)

Supplementary file4 (MOV 4708 KB)

Supplementary file5 (MOV 3081 KB)

Supplementary file1 (PDF 386 KB)

## Data Availability

No datasets were generated or analysed during the current study.
