# Supplementary material for: Computational construction and design optimization of a novel tri-tube heart valve
Source: Biomech Model Mechanobiol. 2025 May 26;24(3):1103–21. doi: 10.1007/s10237-025-01956-5 (PMC12162730; doi:10.1007/s10237-025-01956-5)
Supplement: Supplementary file 6 — Supplementary file1 (PDF 386 KB) [file 10237_2025_1956_MOESM6_ESM.pdf]

Supplementary Information

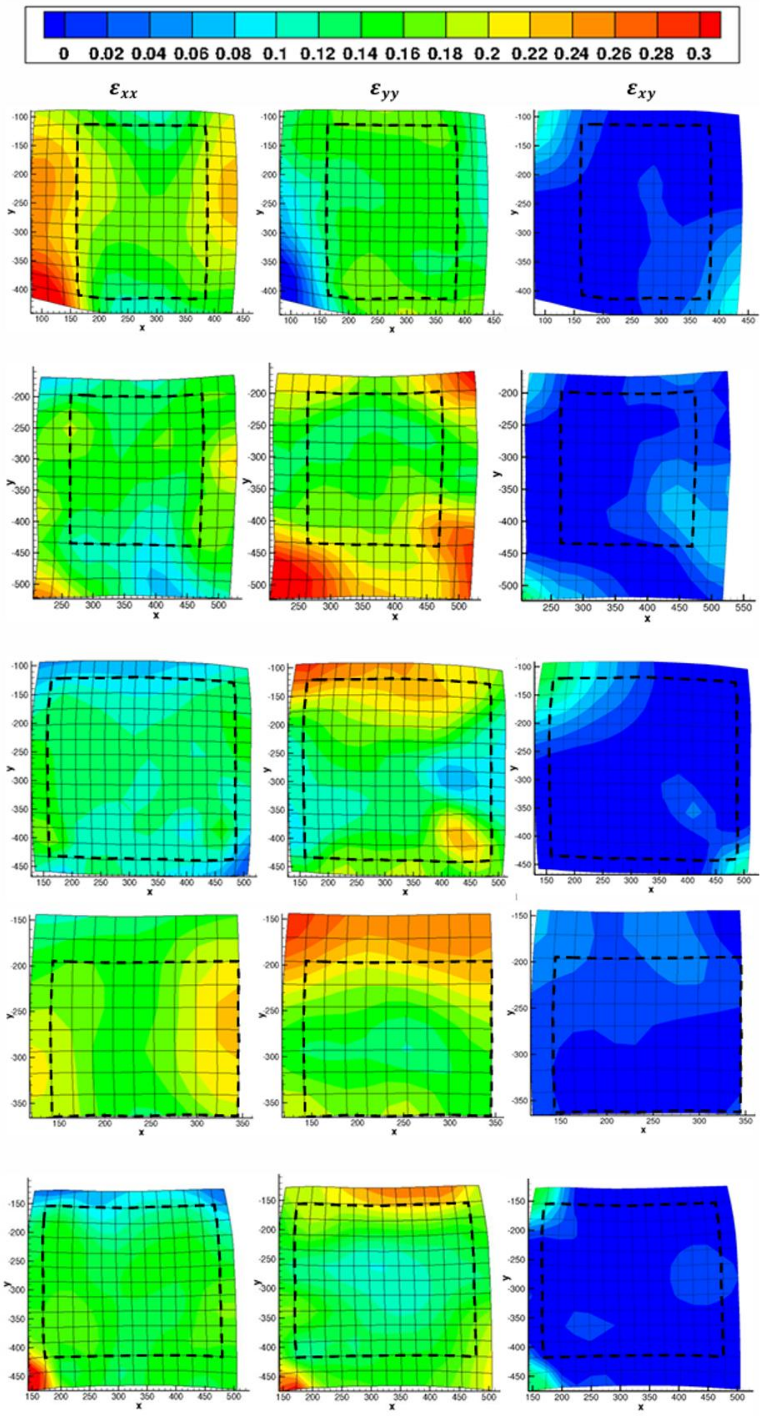

Fig. 10 Local strains obtained during equi-biaixal testing for the five samples tested. From left to right columns: the normal strain in the x direction, the normal strain in the y direction, and the shear strain. Rectangular subregions shown with the dashed black lines with relatively uniform normal strain distribution and minimal shear strains were selected for generating the experimental stress-strain tension curve.

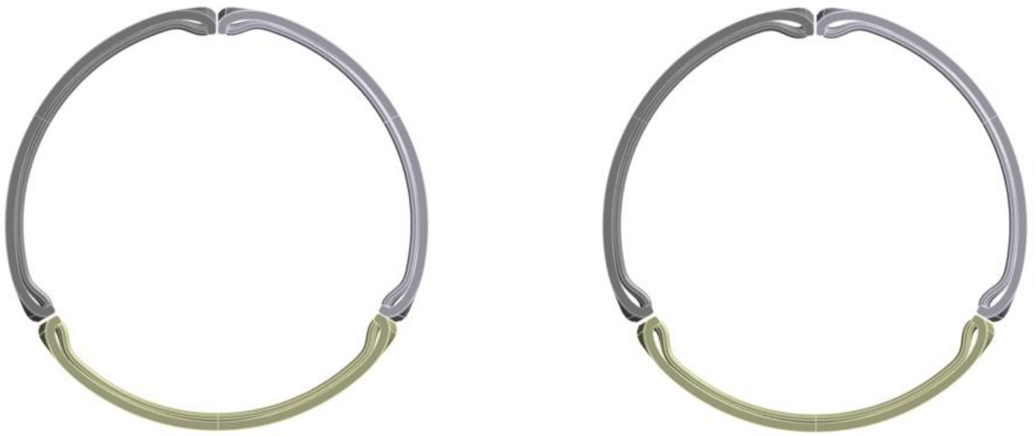

Fig. 11 Constructed valve using different HYPERFOAM fit. Left: HYPERFOAM with fit focusing on compression data (purple curve in Fig. 3b) Right HYPERFOAM with fit including averaged tension data (orange curve in Fig. 3b), using increased pressure to flatten the tube.

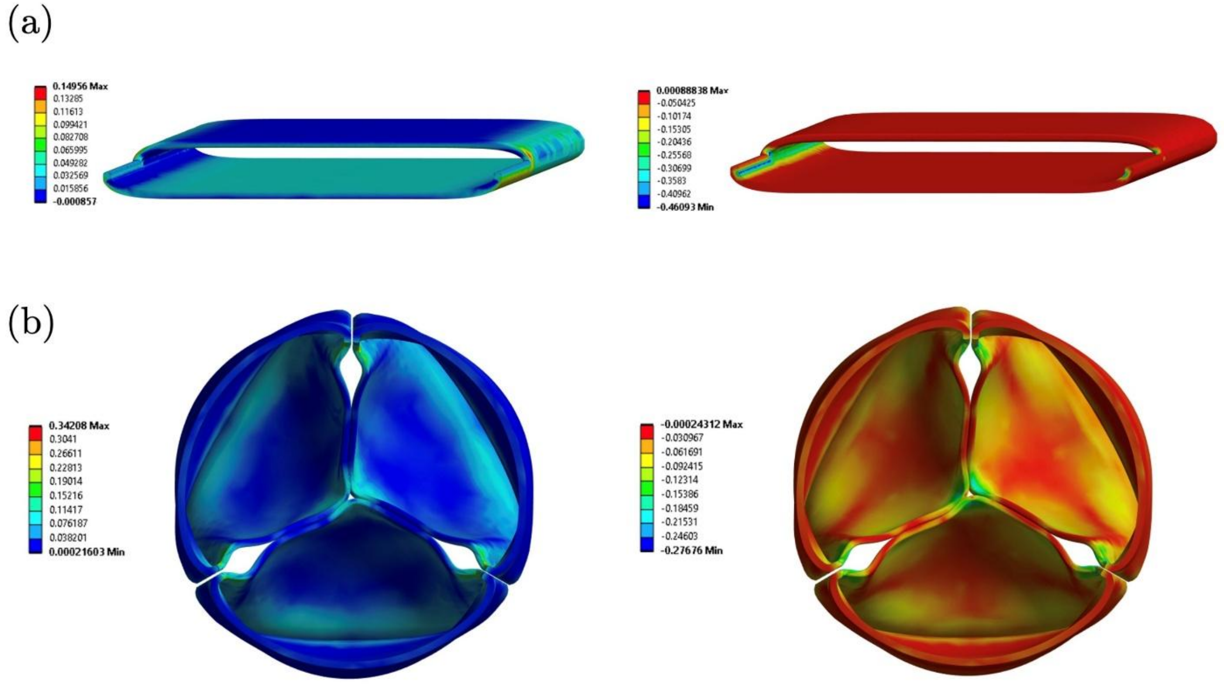

Fig. 12 Maximum (left column) and minimum (right column) principal strain for (a) a snapshot of a flattened tube during valve construction and (b) a snapshot during valve closure simulation.
